# Non-Destructive Assessment of Citrus Mastication Quality Using Magnetic Induction Tomography and Machine Learning

**DOI:** 10.3390/plants15213276

**Published:** 2026-10-23

**Authors:** Yihang Zhu, Zhenxuan Li, Baoliang Xie, Yiying Zhao, Yun Xiang, Xiaobin Zhang

**Affiliations:** 1 Institute of Digital Agriculture, Zhejiang Academy of Agricultural Sciences, Hangzhou 310021, China; zhuyh@zaas.ac.cn (Y.Z.); m15858327936@163.com (B.X.); zhaoyy@zju.edu.cn (Y.Z.); 2 Institute of Cyberspace Security, Zhejiang University of Technology, Hangzhou 310023, China; m13858437607@163.com (Z.L.); xiangyun@zjut.edu.cn (Y.X.); 3 Binjiang Institute of Artificial Intelligence, Zhejiang University of Technology, Hangzhou 310056, China

**Keywords:** magnetic induction tomography (MIT), citrus fruit, mastication quality, non-destructive detection, machine learning

## Abstract

Mastication quality is a key determinant of citrus eating quality, but conventional sensory evaluation and instrumental texture analysis are subjective, destructive, or time-consuming. This study combined magnetic induction tomography (MIT) with machine learning to assess citrus mastication quality non-destructively. MIT signals were acquired from 300 ‘Miyagawa’ satsuma mandarin fruits at 21 frequencies (15–35 MHz). Puncture force, shear force, and chewiness were measured instrumentally. These measurements and sensory scores were integrated into an Analytic Hierarchy Process (AHP)-weighted comprehensive mastication score (0–10), which was also divided into four quality grades (A–D). Feature selection ranked raw features by univariate linear-regression F-test *p*-values for the regression targets or absolute Kendall’s τ values for the classification target. The top 5% of ranked features were retained, and PCA was then fitted to the retained features. Feature distributions were summarized using pooled out-of-fold predictions from nested cross-validation, with five-fold cross-validation used only for inner Bayesian hyperparameter tuning. SVM achieved the highest R^2^ for puncture force (0.825) and shear force (0.762), Random Forest achieved the highest R^2^ for chewiness (0.849) and the highest grade-classification accuracy (64.3%) and macro-averaged AUC (0.871), and XGBoost achieved the highest R^2^ for the comprehensive mastication score (0.835). These results demonstrate the feasibility of combining MIT with machine learning for non-destructive assessment of ‘Miyagawa’ satsuma mandarin citrus mastication quality and identify frequency–spatial patterns that may guide sensor optimization and future grading applications in other citrus fruits.

## 1. Introduction

Mastication quality is a major commercial trait of fresh-market citrus because it directly influences eating experience and market acceptance [1]. Fruit with good mastication quality has juice sacs that rupture readily and segment membranes that disintegrate rapidly, producing a delicate, juicy mouthfeel with little residue [2]. By contrast, fruit with poor mastication quality has thick, tough segment membranes and coarse fibers, leaving substantial residue after chewing and reducing eating quality [3]. As the fresh-citrus market expands, mastication quality has become an important criterion for cultivar selection, postharvest grading, and commercial distribution of cultivars such as ‘Shatangju’ mandarin, ‘Orah’ tangor, and ‘Miyagawa’ satsuma. These satsuma mandarins are characterized by their easy-to-peel rind and typically low seed content, making them suitable for fresh consumption. Their taxonomic classification has varied among citrus classification systems, and their genetic origin has been described as involving mandarin and pomelo ancestry.

Citrus mastication quality is currently evaluated mainly by instrumental texture analysis and trained sensory panels [4]. Texture analyzers objectively measure the fracture properties of segment membranes and juice sacs through puncture force, shear force, and chewiness, with satisfactory repeatability; while trained panels provide a broader assessment of mouthfeel [5]. However, instrumental measurements are destructive, so tested fruit loses its commercial value. The segment-by-segment protocol is also time-consuming for large-scale grading. Sensory evaluation is subjective and difficult to standardize because panelist condition and experience can affect the results, thereby reducing reproducibility [6]. Rapid, objective, and non-destructive methods are therefore needed for citrus mastication quality assessment.

Non-destructive testing (NDT) provides potential tools for rapid, batch-scale evaluation of internal fruit quality. Common approaches include near-infrared spectroscopy (NIR), X-ray computed tomography (X-ray CT), ultrasonics, and electrical impedance spectroscopy (EIS) [7]. NIR is widely used to determine sugar and acid contents, but its limited penetration depth reduces sensitivity to internal fibrous structures [8]. X-ray CT provides three-dimensional structural information, although its high cost and radiation-safety requirements limit use in high-throughput production lines [9]. Ultrasonic methods are sensitive to internal voids and density heterogeneity but have limited resolution for the fine fibrous architecture of citrus segment membranes [10]. EIS responds to the dielectric properties of biological tissues but generally requires contact electrodes, which complicates fully non-destructive measurement [11]. Consequently, existing NDT methods remain inadequate for assessing citrus-specific fibrous texture and segment-membrane characteristics.

Magnetic induction tomography (MIT) is a non-contact imaging technique based on electromagnetic induction and has shown promise for characterizing biological tissues [12]. An excitation coil generates an alternating magnetic field that induces eddy currents in the object. The magnitude and spatial distribution of these currents depend on electrical conductivity, magnetic permeability, and permittivity. Receiving coils detect the resulting secondary magnetic field, and signal processing and image reconstruction are then used to estimate the internal electromagnetic-property distribution [13]. MIT offers several advantages, including non-contact operation, no ionizing radiation, and no requirement for coupling media. Its application to citrus mastication quality is supported by the contrasting tissue structure, water content, and cellulose content of segment membranes and juice sacs, which create differences in dielectric properties, particularly electrical conductivity [2]. Fruit with good mastication quality generally has thinner segment membranes and more easily ruptured juice sacs, whereas poor-quality fruit has thicker membranes and denser fibrous tissue. These structural differences may alter local conductivity and permittivity, generating detectable patterns in the received MIT signals and providing a basis for quantitative, non-destructive assessment [14].

Machine learning is well suited to extracting patterns from high-dimensional sensing data and has been increasingly applied to non-destructive quality assessment of fruit and vegetables [15]. In EIS studies, combinations of principal component analysis (PCA) and support vector machines (SVMs) have been used to discriminate fruit texture and ripeness [16]. In near-infrared and hyperspectral imaging, convolutional neural networks and ensemble methods can extract quality-related information from thousands of wavelength channels [17]. MIT likewise generates high-dimensional data. Projection of signals from several excitation frequencies onto hundreds of spatial elements produces tens of thousands of features, and the resulting frequency–spatial interactions are difficult to model with conventional linear regression [18]. For such high-dimensional, low-sample-size data, a typical workflow combines statistical screening, dimensionality reduction, and nonlinear modeling. Noise features can be removed using analysis of variance (ANOVA) or Kendall rank correlation, PCA can reduce dimensionality and multicollinearity, and ensemble algorithms such as XGBoost and Random Forest can model nonlinear relationships [19]. Similar workflows have been used for EIS, electronic-nose, and hyperspectral data from fruit and vegetables [20], providing a methodological basis for integrating MIT with machine learning.

Accordingly, the primary objective of this study was to investigate the feasibility of combining magnetic induction tomography (MIT) with machine learning to predict mastication-related quality in ‘Miyagawa’ satsuma mandarins. Specifically, the objectives were to (1) characterize the frequency-dependent and spatial distributions of informative MIT features associated with fruit texture; (2) evaluate and compare the predictive performance of different machine-learning models for estimating puncture force, shear force, chewiness, and the AHP-derived comprehensive mastication quality score, as well as classifying fruit into mastication quality grades, as a complementary analysis. The study provides a proof of concept using a single satsuma mandarin cultivar, with broader applicability to other citrus cultivars and species requiring further validation.

## 2. Materials and Methods

### 2.1. Citrus Samples

The study used ‘Miyagawa’ satsuma mandarin (*Citrus unshiu* ‘Miyagawa’), a widely cultivated fresh-market cultivar in eastern and southwestern China. The fruit is typically oblate, with a thin, easily peeled rind and well-developed juice sacs. Its pulp shows natural variation in mastication quality. Sample fruits were harvested from an orchard within a narrow period after physiological maturity. This sampling strategy maintained a relatively uniform production background and maturity while preserving natural variation in mastication quality. After harvest, the fruits were stored at 20 ± 2 °C in a ventilated, dark environment, and all measurements were completed within several days to minimize tissue softening and changes in dielectric properties. Fruit with skin damage, visible decay, or mechanical injury were excluded. After applying these exclusion criteria, 300 fruits were retained for subsequent measurement and analysis. Before MIT measurement, the fruit diameter of all samples was measured, and all samples were equilibrated to room temperature to minimize the influence of temperature gradients on the signals.

### 2.2. Magnetic Induction Tomography Measurement

#### 2.2.1. Measurement System

An MT-200 magnetic induction tomography system (Hangzhou Yongchuan Technology Co., Ltd., Hangzhou, China; hereafter, the MIT system) was used for non-destructive fruit scanning. The system comprises an excitation/receiving coil array with 16 coils evenly distributed, a multi-frequency signal generator, and a data acquisition and control unit. The excitation coil array with a diameter of 20 cm generates an alternating magnetic field over the selected frequency range and induces eddy currents in the fruit. The receiving coil detects the resulting secondary magnetic field in each scanning iteration. After analog-to-digital conversion and signal stabilization, the system outputs amplitude and phase data for each receiving channel, then reconstructs the induced-voltage signals employing data in all scanning iterations. Repeated scan or repositioning is not required according to the system manual, because one coil acts as receiving channel and the remaining coils as excitation channels in each scanning iteration. In multi-frequency mode, a complete scan acquires induced-voltage signals at 21 frequencies from 15 to 35 MHz, in 1 MHz increments, for subsequent feature extraction.

#### 2.2.2. Scanning Protocol

The system was placed on a horizontal surface composed of non-metals, such as cardboard, to avoid the impact of metal on electromagnetic signals. Before measurement, a plastic vessel filled with purified water was placed at the center of the coil array, and a reference scan was acquired for baseline calibration. Each fruit was then positioned at the center of the coil array with its equatorial plane parallel to the coil plane to maintain consistent geometry. A complete multi-frequency scan required approximately 2 to 3 min, depending on frequency switching and signal stabilization. The fruit remained stationary and no contact pressure was applied, so the procedure was fully non-contact and non-destructive. Raw signals were processed with the system software using noise filtering, baseline correction, channel balancing, and reference calibration. The signal-to-noise ratio (SNR) at each measurement frequency is provided in Supplementary Table S1.

#### 2.2.3. Signal Feature Structure

The preprocessed MIT signals were reconstructed and projected onto a spatial mesh. Reconstruction was based on the finite element method (FEM), which divides the imaging domain into non-overlapping triangular elements and approximates the electromagnetic governing equations within each element [21]. Following the coarse-mesh scheme and analysis process of the MITNet framework proposed by [22,23], the signal distribution at each frequency was reconstructed on a triangular mesh containing 1024 elements. The data for each sample were therefore represented by a 21 × 1024 matrix, corresponding to 21 excitation frequencies and 1024 spatial elements. Vectorization produced 21,504 raw features per sample. These features captured spatially resolved electromagnetic responses across the frequency range and were used for subsequent feature engineering and machine learning.

### 2.3. Mastication Quality Evaluation

#### 2.3.1. Instrumental Texture Measurement

Destructive mechanical testing was conducted immediately after the MIT scan to minimize the interval between non-destructive measurement and reference-property assessment. A FirmTech eleven texture analyzer (Umweltanalytische Produkte GmbH, Cottbus, Germany) was used to measure puncture force, shear force, and chewiness for each sample. Fruits were manually separated into segments, and intact, undamaged segments were placed on the sample platform. For puncture testing, a pin probe (2 mm width) penetrated the segment flesh vertically at 2 mm s^−1^ to a predetermined depth (20 mm), and the puncture force was recorded in gram-force (gf). For repeated-compression testing, the same probe penetrated the segment flesh at the same speed to the same predetermined depth (20 mm) twice, and the second peak force recorded times the ratio of the two peaks’ time intervals during this procedure was used as the operational texture indicator referred to as chewiness (gf) in this study. For shear testing, the blade probe (2 mm width) cut transversely through the segment at the same speed, and the shear force was recorded in gf. The measurements were conducted using the instrument’s plate with flat base holding the segments. Three segments from each fruit were tested, and their mean was used as the representative value to reduce the effect of natural variation among segments. Puncture force mainly reflects segment-membrane toughness and juice-sac rupture resistance, shear force reflects the cutting resistance of fibrous structures, and chewiness represents the integrated mechanical response during repeated mastication. Together, these variables describe the mechanical basis of citrus mastication quality. All the measurements and the fruit diameter are listed in Supplementary Table S2.

#### 2.3.2. Comprehensive Mastication Score

A single instrumental texture parameter is insufficient to comprehensively capture the multidimensional characteristics of mastication quality as perceived in actual consumer sensory experience. To address this limitation, this study employed the Analytic Hierarchy Process (AHP) to fuse instrumental texture measurements with expert sensory evaluation scores through weighted combination, thereby constructing a comprehensive mastication score, referring to Baviera-Puig et al. [24].

Standardized training of panelists was conducted in accordance with the GBT29605-2013 standard one day before the evaluation. Citrus fruits with known mastication levels (not necessarily the same cultivar) were used for training. Panel calibration was verified dynamically; panelists were cleared for formal evaluation only when individual scoring variance across blind duplicate reference samples achieved a coefficient of variation (CV) of less than 20%. Each fruit typically contained approximately 9–12 segments. Three segments were used for instrumental texture testing, while the remaining segments from the same fruit were used for sensory evaluation, which was conducted by a professionally trained sensory panel. During the evaluation, ten panelists assessed each fruit sample following a standardized protocol, samples were randomly coded, and purified drinking water was used to clean the palate between samples. The sensory scores (1–10, in 1 increments) were then averaged from all panelists’ ratings and aggregated into a composite sensory score. The AHP weight matrix was constructed through pairwise comparison judgment matrices, and the rationality of weight allocation was verified via consistency ratio checks (CR < 0.1).

Ultimately, puncture force, shear force, chewiness, and sensory scores were linearly weighted according to the AHP-derived weights to yield a comprehensive mastication score for each sample. The final AHP-derived weights and correlation with comprehensive mastication score for puncture force, shear force, chewiness, and the sensory score were presented in Supplementary Table S3. The consistency ratio of the pairwise-comparison matrix was CR = 0.054. Because higher puncture force, shear force, and chewiness values were interpreted as indicating greater mechanical resistance and poorer mastication quality, their directions were inverted before aggregation. The final comprehensive score was oriented so that higher values represented better mastication quality. The score was standardized to a 0–10 scale using all 300 samples, with higher scores indicating superior mastication quality.

#### 2.3.3. Mastication Quality Grade Classification

For exploratory classification, fruits were assigned to four quality grades (A–D) according to the quartiles of the AHP-derived comprehensive mastication score. This quartile-based scheme was adopted to discretize the continuous score into four relative quality categories and to provide a classification task complementary to continuous score prediction. Samples scoring above the third quartile (Q3, top 25%) were assigned to Grade A; those between Q2 and Q3 to Grade B; those between Q1 and Q2 to Grade C; and those below the first quartile (Q1, bottom 25%) to Grade D. This scheme provides approximately balanced class labels for subsequent classification model training and evaluation.

### 2.4. Feature Engineering

#### 2.4.1. Frequency Band Feature Importance Analysis

Vectorization of the raw feature matrix produced 21,504 variables, and direct model input would increase dimensionality and introduce redundant noise. The raw dataset was organized as a matrix of 300 samples × 21,504 features, with each sample represented by a 21 × 1024 matrix corresponding to 21 excitation frequencies (15–35 MHz) and 1024 spatial elements. For descriptive analysis of the frequency–spatial distribution of signal variance, PCA was applied to the full 300 × 21,504 matrix, and the resulting feature loadings were mapped back to the original feature space. The 21,504 features were then grouped into 21 frequency-specific blocks, each containing 1024 spatial features. For each frequency, the mean absolute loading across its 1024 spatial features was calculated to summarize the distribution of PCA loadings associated with that frequency. Thus, the 1024 elements represent the spatial features within each frequency, rather than a separate dataset used for PCA. For predictive modeling, feature ranking and selection were conducted separately for each target, followed by PCA fitted to the selected features.

#### 2.4.2. Feature Selection

**Figure 1:**
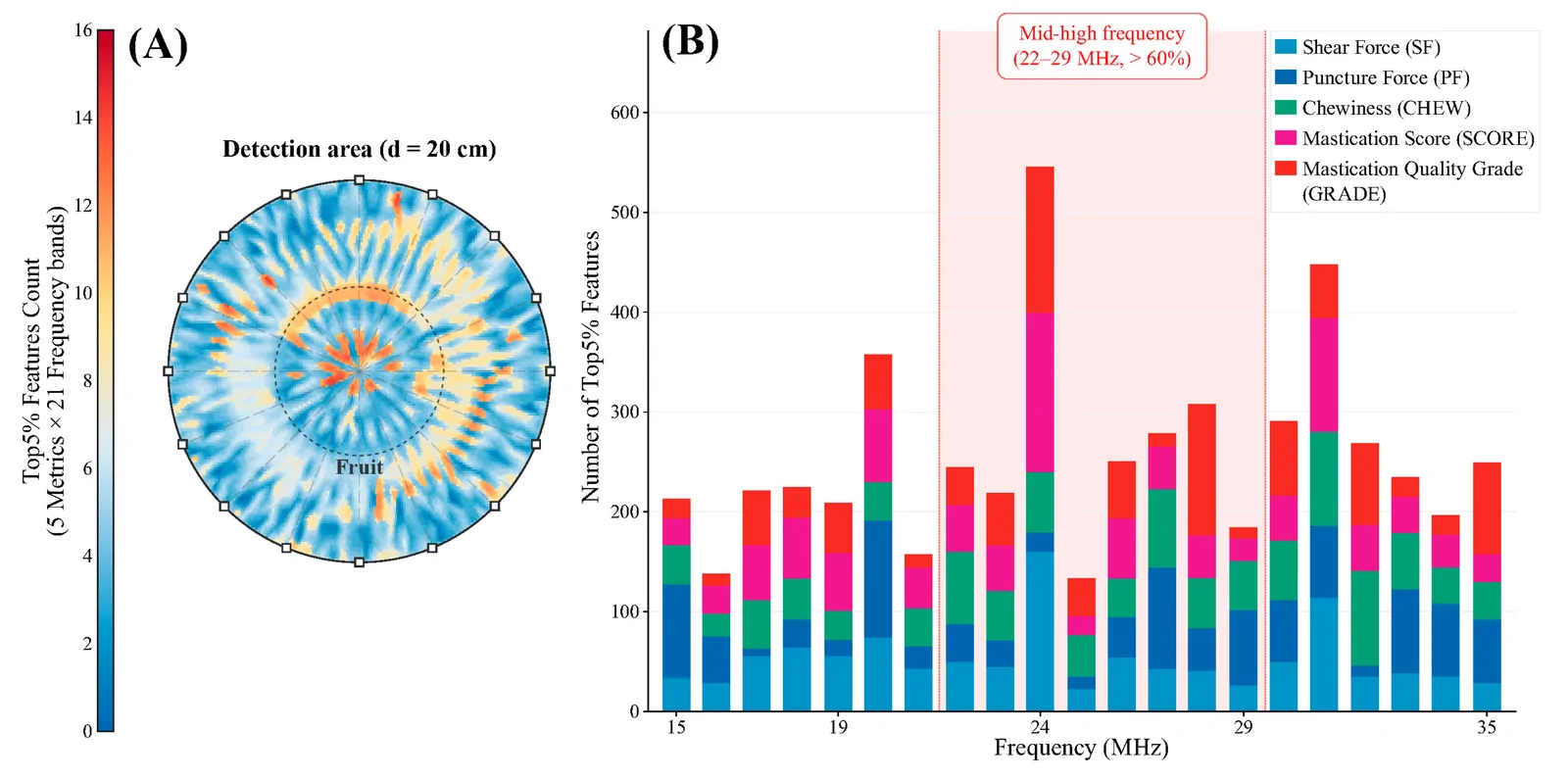
Frequency-band feature importance: (**A**) Spatial heatmap distributions and (**B**) stacked bar chart of selected-feature counts across 21 frequency bands (15–35 MHz). The small squares on the circumference and the dashed circle in the middle in (**A**) indicate the coil array of the MIT device and the approximate fruit positioning area, respectively.

**Figure 2:**
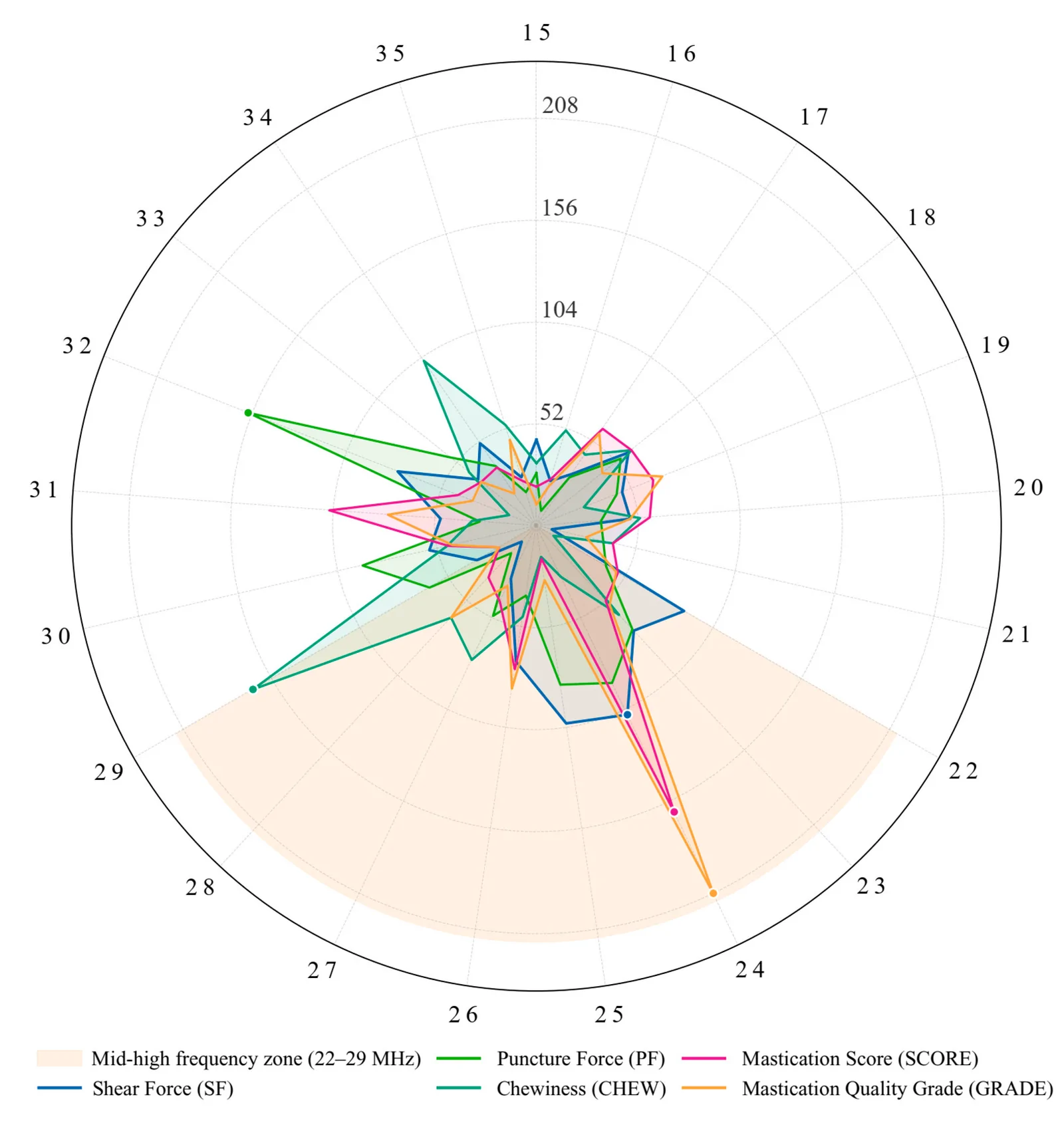
Radar chart of selected top 5% feature counts for five mastication-quality-related targets across 21 frequency bands (15–35 MHz), with peak annotations.

**Figure 3:**
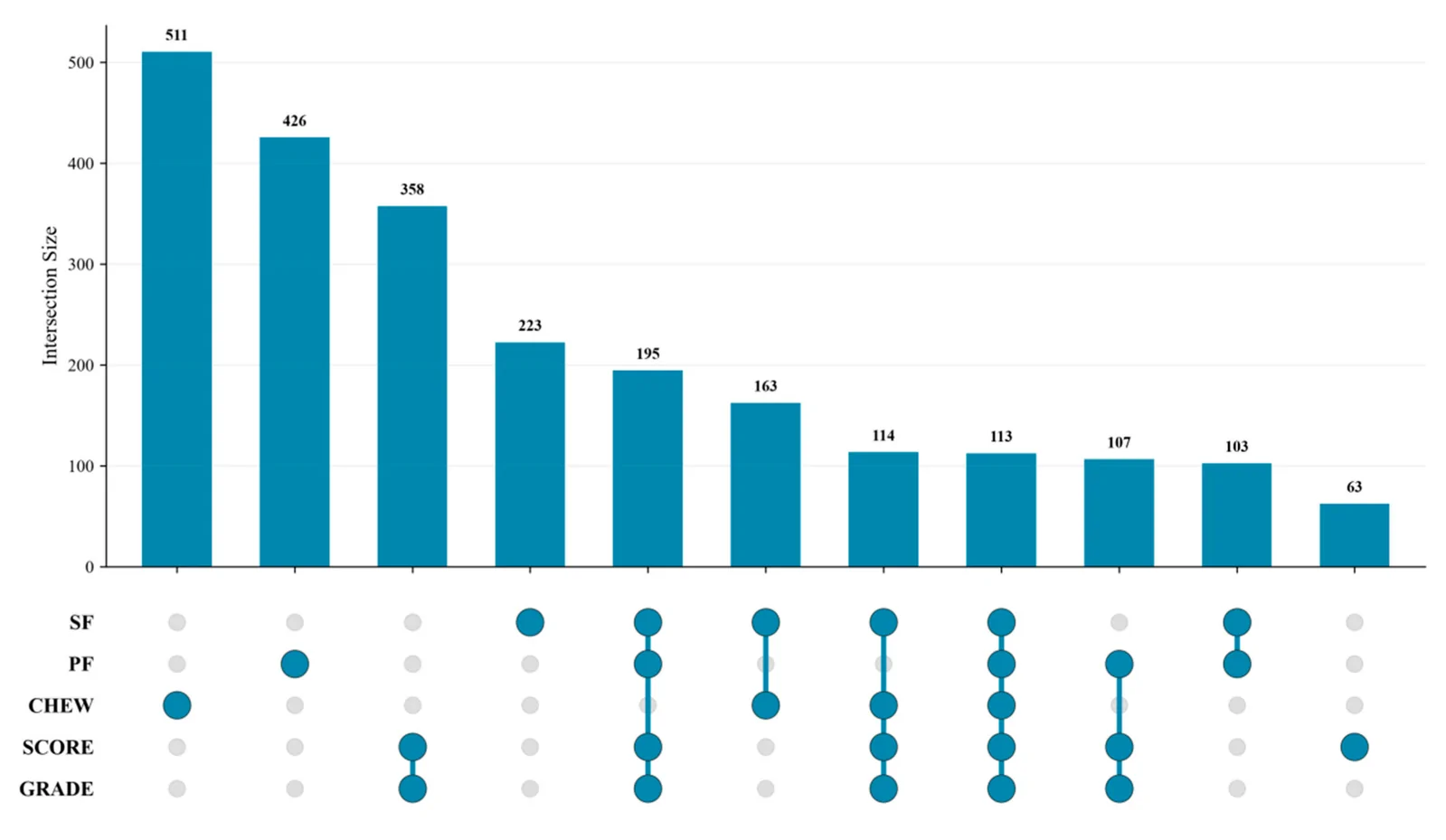
UpSet intersection visualization: feature intersection patterns of five mastication quality targets (SF/PF/CHEW/SCORE/GRADE) at the top 5% significance threshold.

Statistical screening was performed separately for puncture force, shear force, chewiness, the comprehensive mastication score, and mastication quality grade. For the four regression targets, univariate linear-regression F-tests were used to obtain *p*-values for ranking. For the classification target, features were ranked by the absolute value of Kendall’s τ. In both cases, feature selection reduced the original 21,504 features to 1075 features, corresponding to the top 5% of features ranked by importance. These selected features were used for the subsequent PCA and machine-learning workflow. Figure 1, Figure 2 and Figure 3 present the feature distributions obtained from the full dataset to provide a descriptive overview of the selected features and their frequency–spatial characteristics. For predictive model evaluation, feature selection was performed independently within each cross-validation training fold, and the selected features from that fold were subsequently used for PCA and model training.

#### 2.4.3. Dimensionality Reduction

The selected feature sets may still contain multicollinearity. PCA was therefore applied to the selected features to reduce dimensionality while preserving the majority of the feature variance. The number of principal components was determined by retaining the smallest number of components required to explain at least 95% of the cumulative variance. During cross-validation, PCA was fitted independently within each training fold, and the number of retained components was determined separately for each fold. The fitted PCA transformation was then applied to the corresponding held-out data. Accordingly, the number of retained components could vary across folds. These components formed the final model inputs and reduced redundancy while preserving most of the information in the selected features.

### 2.5. Machine Learning Models

#### 2.5.1. Model Selection

XGBoost, Random Forest (RF), and an SVM framework were used for four regression tasks, namely puncture force, shear force, chewiness, and comprehensive mastication score, and one classification task, namely mastication quality grade. R packages *xgboost* 3.0.1, *randomForest* 4.7, *e1071* 1.7 were used for the corresponding framework. Within the SVM framework, support vector regression (SVR) was used for regression and support vector classification (SVC) was used for classification. All models were trained on the same data and evaluated with the same validation protocol to provide direct performance comparisons.

XGBoost builds an ensemble of decision trees through gradient boosting and uses L1 and L2 regularization to limit overfitting. It can handle high-dimensional sparse features and nonlinear interactions, making it suitable for MIT data. RF combines independently trained trees through bagging and is robust to noise and outliers. Within the SVM framework, SVR and SVC use a radial basis function (RBF) kernel to map features into a higher-dimensional space and often generalize well in small-sample, high-dimensional settings.

#### 2.5.2. Hyperparameter Optimization

Bayesian optimization was employed implemented in R using the *rBayesianOptimization* package (version 1.2.2) for systematic tuning of hyperparameters for each model. For XGBoost, the principal optimized parameters included: maximum tree depth (max_depth: 2–10), learning rate (learning_rate: 0.01–0.3), subsample ratio (subsample: 0.6–1.0), column sampling ratio (colsample_bytree: 0.6–1.0), and regularization coefficients (lambda, alpha). For RF, the optimized parameters included: number of estimators (n_estimators: 50–500) and maximum number of features (max_features). For SVR and SVC, the optimized parameters included penalty coefficient C (0.01–1000) and RBF kernel bandwidth (10^−5^–1). The mean objective metric from five-fold cross-validation was used only within each outer training fold as the inner Bayesian-optimization objective, with 50 iterations used to search the hyperparameter space.

#### 2.5.3. Cross-Validation Strategy

Model performance was evaluated by outer 10-fold cross-validation. The 300 samples were randomly divided into 10 approximately equal folds. In each outer iteration, nine folds were used for model development and one was held out for testing. Within the outer training data, target-specific feature ranking, selection of the top 5%, PCA fitting, and five-fold Bayesian hyperparameter tuning were performed independently to prevent data leakage. Specifically, feature importance ranking and feature selection were conducted using only the training data in each fold, and PCA was fitted to the selected training features. The fitted preprocessing transformations were then applied to the corresponding held-out fold before model evaluation. Each sample therefore contributed one out-of-fold prediction, and performance metrics were calculated from the pooled out-of-fold predictions across all 300 samples rather than by averaging fold-wise metrics.

### 2.6. Evaluation Metrics

#### 2.6.1. Regression Evaluation Metrics

Performance on the four regression tasks, puncture force, shear force, chewiness, and comprehensive mastication score, was evaluated using root mean squared error (RMSE), relative root mean square error (RRMSE), and the coefficient of determination (R^2^). RMSE and RRMSE are defined as:

$$RMSE=\sqrt{\frac{1}{n}\sum_{i=1}^{n}(y_{i}-{\hat{y}}_{i}{)}^{2}}$$

$$\mathrm{RRMSE}=\frac{RMSE}{\bar{y}}\times 100\%$$

where $y_{i}$ denotes the observed value of the *i*-th sample, ${\hat{y}}_{i}$ denotes the model-predicted value, $\bar{y}$ denotes the mean of all observed values, and n denotes the total number of samples. RRMSE normalizes the prediction error by the mean, thereby eliminating the influence of scale differences across indicators and facilitating cross-indicator comparison of prediction accuracy; lower RRMSE values indicate higher prediction accuracy. R^2^ reflects the proportion of variance in the observed values explained by the model predictions; values approaching 1 indicate superior goodness of fit.

#### 2.6.2. Classification Evaluation Metrics

For the four-class classification task (mastication quality grades A/B/C/D), overall accuracy, the area under the receiver operating characteristic curve (AUC), and the confusion matrix were employed for comprehensive evaluation. AUC was computed for each grade using the one-versus-rest strategy to obtain per-class binary AUC values, which were subsequently aggregated via macro-averaging to yield the overall AUC. The confusion matrix was examined to identify the most frequent misclassifications between the four grades (A–D). In particular, the analysis considered which grades were most often incorrectly assigned to other grades. These patterns were used to characterize the classification errors, rather than to imply an established ordering or mechanism underlying the misclassifications.

## 3. Results

### 3.1. MIT Signal Characteristics and Feature Importance Analysis

#### 3.1.1. Frequency–Spatial Distribution Patterns of Feature Importance

Feature-importance analysis was performed on the MIT matrices from the 300 samples using PCA back-projection for descriptive frequency–spatial analysis, target-specific top 5% feature selection, and the nested validation protocol described above for XGBoost, RF, and the SVM framework. The 21 excitation frequencies (15–35 MHz) contributed unevenly to the five mastication-quality-related targets. Across the 1024-element FEM mesh, high-importance regions were concentrated near the boundary of the fruit placement area, corresponding to locations where segment membranes and juice sacs alternate in the transverse section. This spatial pattern suggests that MIT is sensitive to dielectric contrast at the segment membrane–juice sac interface.

Figure 1A presents the feature-importance heatmap across 1024 spatial nodes on the FEM-reconstructed mesh, with color encoding the aggregate count of the five mastication-quality-related targets for which each feature was selected in the top 5%; the solid large circle delineates the sensing-domain boundary (20 cm diameter), with 16 square markers along the boundary indicating coil positions, and the dashed small circle denoting the fruit placement position.

Figure 1B displays a stacked bar chart across the 21 excitation-frequency channels (15–35 MHz, at 1 MHz intervals), with the five mastication-quality-related targets color-coded and stacked, and the mid-to-high frequency region (22–29 MHz) highlighted by red shading.

Examined from the frequency dimension, the mean feature importance of mid-to-high frequency bands (22–29 MHz) was markedly higher than that of low-frequency bands (15–21 MHz). Among the 21 frequency channels, channels 8 through 15 cumulatively accounted for over 60% of the total feature importance, whereas the lowest five channels contributed less than 15%, indicating that low-frequency signals provided limited discrimination for internal fibrous texture variations in citrus fruits. This pattern may be related to differences in dielectric responses between segment-membrane fibrous structures and the liquid matrix of juice sacs under higher-frequency excitation.

#### 3.1.2. Frequency Band Distribution of Top 5% High-Importance Features Across Indicators

Radar charts (Figure 2) were used to show the distribution of the top 5% selected features for puncture force, shear force, chewiness, comprehensive mastication score, and mastication quality grade across the 21 frequencies. The five mastication-quality-related targets showed broadly similar distributions with local differences. Puncture force and shear force had closely aligned profiles and clear peaks at mid-to-high frequencies. The comprehensive score had a somewhat broader distribution, possibly because it incorporated sensory information. Grade classification showed a distribution similar to that of the comprehensive score, with localized increases at several frequencies that may contribute to discrimination near grade boundaries.

Specifically, the polar radar chart displays the selected-feature count distributions of SF (shear force, peak at 32 MHz), PF (puncture force, peak at 29 MHz), CHEW (chewiness, peak at 30 MHz), SCORE (comprehensive mastication score, peak at 26 MHz), and GRADE (mastication quality grade, peak at 28 MHz) across the 21 frequency bands (15–35 MHz). The peak selected-feature importance for SF was concentrated in the 24–32 MHz band, which was broader than the primary interval (22–29 MHz) observed for the other targets. The 24–29 MHz region partially overlapped with the selected-feature distributions of the other targets, whereas the 29–32 MHz region was more specific to SF. The mid-to-high frequency region (22–29 MHz) is highlighted with semi-transparent orange shading, and the peak of each target is annotated with a white-edged circular marker. The legend includes the frequency-band peak information for each target, and the radial axis represents the absolute feature counts for the respective targets.

#### 3.1.3. Feature Overlap Analysis

UpSet analysis compared the top 5% selected-feature sets for the five mastication-quality-related targets, as shown in Figure 3. Each set contained 1075 features, but their intersection patterns differed substantially. CHEW had 511 unique features (47.5%), the largest exclusive proportion, indicating relatively independent MIT information for chewiness. It also shared 564 features (52.5%) with other targets, including 489 (45.5%) with SF. SF and PF had 223 (20.7%) and 426 (39.6%) unique features, respectively, and shared 510 features (47.4%); 103 features (9.6%) were shared only by SF and PF. This overlap indicates partially common dielectric responses at the segment membrane–juice sac interface. SCORE and GRADE shared 963 features (89.6%), the largest overlap, indicating that grade classification largely inherited the signal basis of the AHP-weighted comprehensive score. GRADE had only 15 unique features (1.4% of its set; 0.6% of all selected features), and SCORE had 63 (5.9%), showing that the regression and classification tasks tended to converge in their selected feature subsets.

The UpSet plot shows the 11 largest non-empty intersection combinations among the five target-specific selected-feature sets; smaller non-empty combinations are not displayed. The largest intersection was shared by SCORE and GRADE, with 963 features (89.6% of each set), followed by the intersection of all five targets, with 52 features (4.8% of each set). The pattern indicates strong coupling between the continuous comprehensive score and its derived mastication quality grade, whereas CHEW retained a comparatively independent signal component.

The lower matrix plot employs blue dots to indicate indicators included in each intersection combination and light gray dots to denote those excluded; within each combination, dots are connected by vertical lines to directly visualize the indicator composition of each intersection.

To examine the overlap in informative MIT features across the five prediction targets, the selected features were compared across targets. Although 1075 features were selected for each target, some features were selected for multiple targets. After accounting for overlap, the combined set comprised 2651 target-specific feature instances, which were subsequently analyzed to distinguish features shared across targets from those unique to individual targets. The large overlap between SCORE and GRADE is consistent with their shared AHP-derived origin, whereas the greater number of CHEW-specific features suggests that chewiness captures additional frequency–spatial information. These results provide a basis for target-aware sensor optimization rather than assuming that one common feature subset will be optimal for all outputs.

### 3.2. Model Comparison

Ten-fold cross-validation was employed to systematically compare the performance of XGBoost, Random Forest, and SVM across five prediction tasks. Table 1 summarizes the R^2^ and RRMSE of each model on the regression tasks (puncture force, shear force, chewiness, and comprehensive mastication score), as well as the overall accuracy and macro-averaged AUC on the grade classification task. The results revealed minimal performance differences among the three models across all tasks. SVM marginally outperformed XGBoost and Random Forest on the puncture force and shear force regression tasks, while Random Forest achieved the highest R^2^ (0.849) for chewiness prediction. The three models exhibited the closest performance on the chewiness task (R^2^ difference < 0.01).

In the regression tasks, SVM achieved the highest R^2^ values for puncture force (0.825) and shear force (0.762), slightly surpassing XGBoost and Random Forest, though the differences among the three models were generally small (R^2^ differences typically within 0.04). For comprehensive mastication score prediction, XGBoost attained the highest R^2^ (0.835). On the chewiness prediction task, the three models performed remarkably closely (XGBoost R^2^ = 0.842, Random Forest R^2^ = 0.849, SVM R^2^ = 0.846), with differences of less than 0.01. In terms of RRMSE, SVM yielded the lowest values for the puncture force and shear force tasks, Random Forest achieved the lowest RRMSE for chewiness, and XGBoost obtained the lowest RRMSE for the comprehensive score task. For the grade classification task, Random Forest achieved the highest overall accuracy at 64.3%, exceeding SVM (63.3%) and XGBoost (61.7%) by 1.0 and 2.7 percentage points, respectively. Regarding macro-averaged AUC, Random Forest attained 0.871, followed by XGBoost at 0.861 and SVM at 0.847. The classification performance of all three models was at a moderate level, with confusion between adjacent grades (B and C) constituting the primary source of misclassification.

The three models, SVM, XGBoost, and RF, demonstrated comparable overall prediction performance across the five mastication-quality-related targets. SVM exhibited a slight advantage in the puncture force and shear force regression tasks, while RF achieved the best performance in the classification task. Overall, all three models effectively captured associations between MIT signals and citrus mastication quality under the conditions of this study, and the subsequent analyses are discussed based on their comprehensive performance.

### 3.3. Regression Prediction Performance

#### 3.3.1. Puncture Force Prediction

**Figure 4:**
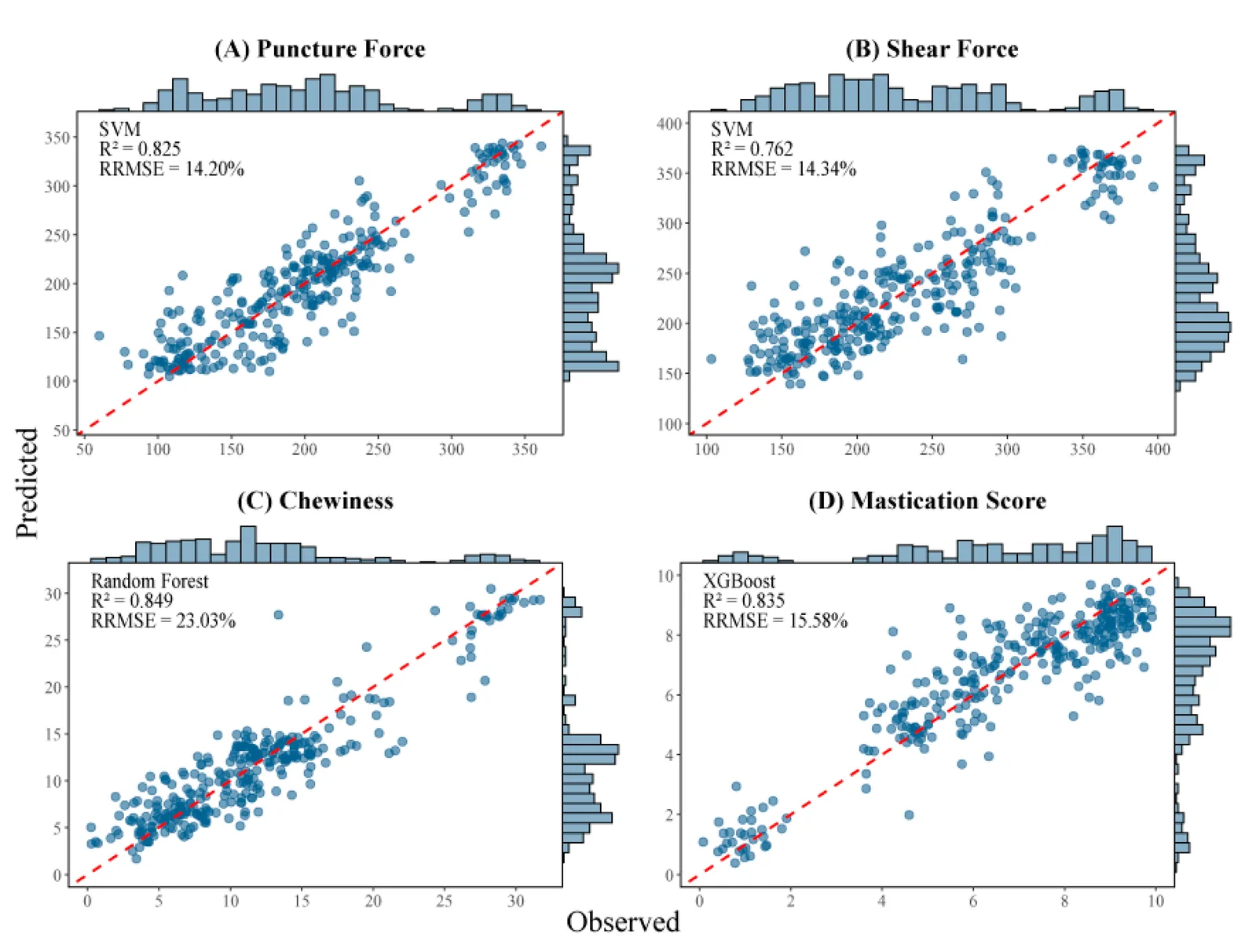
Regression performance for citrus mastication quality traits based on MIT spectral data (10-fold cross-validation).

SVM provided the best puncture-force predictions, with R^2^ = 0.825, RMSE = 28.211 and RRMSE = 0.142 (Table 1; Figure 4A), R^2^, RMSE and RRMSE become 0.808, 28.910 and 0.162, respectively, when the top-right cluster is removed. Predicted and observed values were distributed around the y = x line. RMSE was lowest in the high-force range (>250 g; 17.872) and higher in the low-force range (<250 g; 28.910). The weaker performance at low forces may reflect the thinner segment membranes and lower signal contrast of these fruits. Because puncture force reflects segment-membrane toughness, its predictability supports the ability of MIT to sense relevant mechanical properties without contact. SVM achieved the highest R^2^, followed by RF (0.816) and XGBoost (0.789), but all differences were within 0.04.

#### 3.3.2. Shear Force Prediction

SVM also provided the best shear-force predictions, with R^2^ = 0.762, RMSE = 33.444 and RRMSE = 0.143 (Table 1; Figure 4B), R^2^, RMSE and RRMSE become 0.739, 34.456 and 0.161, respectively, when the top-right cluster is removed. Several samples in the intermediate range (200–280 g) showed large deviations, with a maximum of 106 g. These errors may reflect variation in juice-sac fiber orientation during single-segment testing. Shear force represents the combined cutting resistance of segment-membrane fibers and internal vascular bundles, producing a more complex signal than puncture force and potentially reducing prediction accuracy. SVM achieved the highest R^2^, followed by RF (0.759) and XGBoost (0.729); the differences remained within 0.04.

#### 3.3.3. Chewiness Prediction

RF provided the best chewiness predictions, with R^2^ = 0.849, RMSE = 2.722 and RRMSE = 0.230 (Table 1; Figure 4C), R^2^, RMSE and RRMSE become 0.810, 2.746 and 0.274, respectively, when the top-right cluster is removed. The relatively high RRMSE largely reflects the narrow distribution of measured chewiness values (median ≈ 11; IQR ≈ 6.7–14.5), for which small absolute errors produce larger relative errors. Predicted and observed values were distributed around the y = x line, and errors were smaller at low (<8) and high (>14) values than in the intermediate range. Performance was nearly identical among XGBoost (R^2^ = 0.842), RF (R^2^ = 0.849), and SVM (R^2^ = 0.846), with differences below 0.01. Chewiness integrates segment-membrane rupture, juice-sac fragmentation, and fiber breakdown, and this complex response may explain why tree-based and kernel-based models perform similarly.

#### 3.3.4. Composite Mastication Score Prediction

The comprehensive mastication score was the target most directly aligned with the intended application. XGBoost achieved the best score prediction, with R^2^ = 0.835, RMSE = 1.029 and RRMSE = 0.156 (Table 1; Figure 4D), R^2^, RMSE and RRMSE become 0.809, 1.061 and 0.147, respectively, when the bottom-left cluster is removed. Predicted and observed values were distributed around the y = x line across the 0–10 range, with no clear systematic bias. RMSE was lowest below 3 points (1.005) and higher above 3 points (1.061), possibly because mastication characteristics varied more complexly in the high-score range. XGBoost achieved the highest R^2^, followed closely by RF (0.830) and then SVM (0.785). The 0.005 difference between XGBoost and RF was small, whereas both tree-based models exceeded SVM by more than 0.04. An R^2^ above 0.83 indicates that MIT features can capture substantial population-level variation in the comprehensive score and may support non-destructive assessment as a complement to destructive texture testing and sensory evaluation.

To examine the potential association between fruit size and the target variables, Pearson correlation analysis was conducted using the measured fruit diameter. The correlation coefficient was (r = 0.172), indicating a negligible linear association for the analyzed variables. This result suggests that fruit diameter alone is unlikely to explain the observed variation in the target measurements. However, the correlation does not exclude potential effects of fruit size on the MIT signals or establish that the predictive models are independent of size-related influences.

### 3.4. Classification Performance

#### 3.4.1. Confusion Matrix and Grade Discrimination

**Figure 5:**
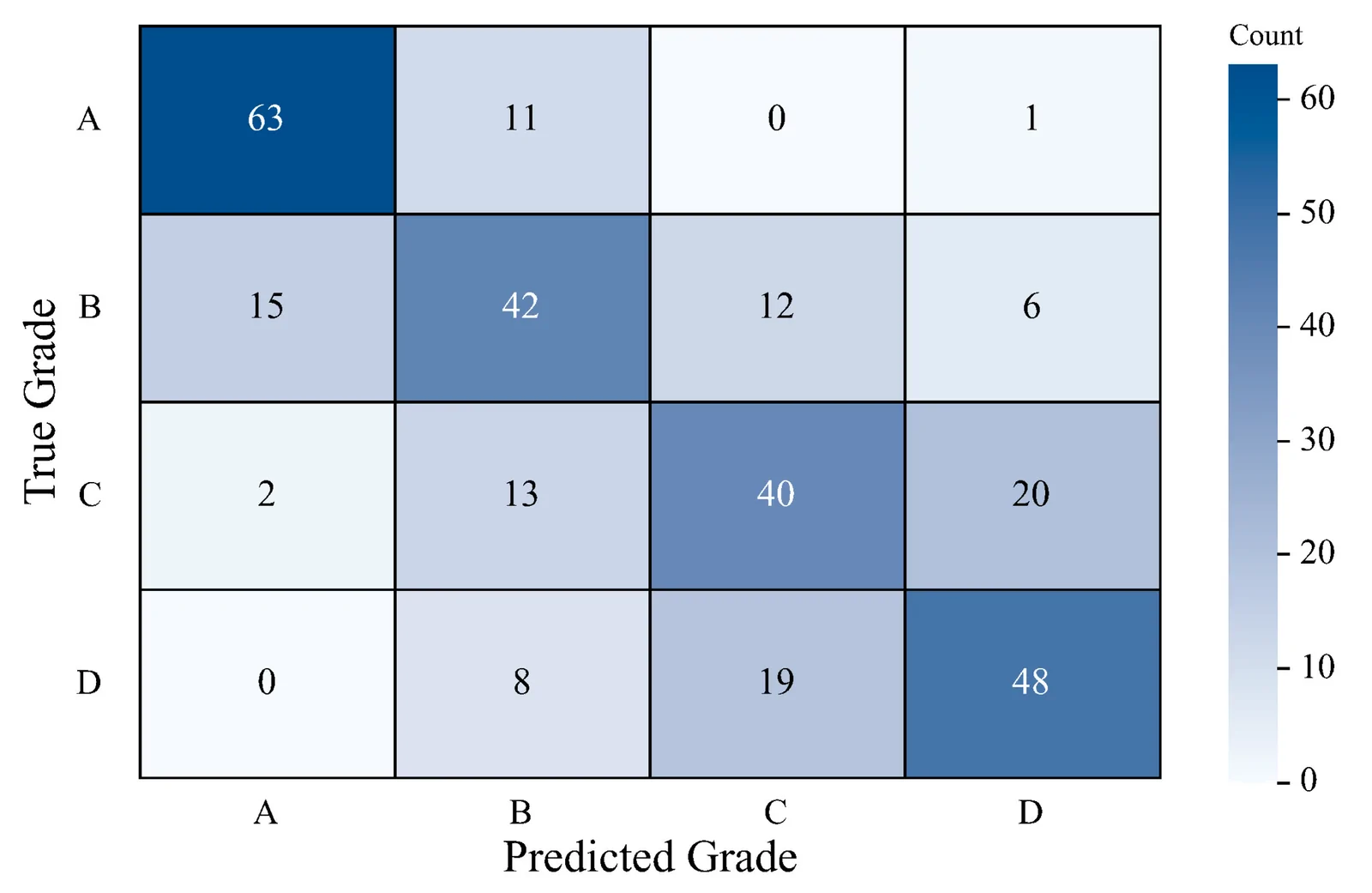
Confusion matrix for citrus grade classification based on MIT spectral data (RF, 10-fold cross-validation).

The Random Forest four-class classification model achieved the best overall accuracy of 64.3% and macro-average AUC of 0.871 in the 10-fold cross-validation across 300 samples. The confusion matrix (Figure 5) revealed varying discrimination accuracies among the four grades: grade A yielded an accuracy of 84.0%, and grade D yielded 53.3%, both exceeding the overall average; grades B and C achieved accuracies of 56.0% and 64.0%, respectively, both below those of the two terminal grades. Confusion between adjacent grades (e.g., B and C) constituted the primary source of misclassification, whereas misclassifications spanning two or more grades (e.g., A and C, B and D) accounted for a negligible proportion, collectively less than 5%, indicating that the model possessed a certain degree of directional discrimination capability along the mastication quality gradient. The performance differences among the three models were marginal, all falling within the range of 61–65%.

#### 3.4.2. Per-Class AUC Analysis

For RF, one-versus-rest AUC values were 0.958 for Grade A, 0.828 for Grade B, 0.813 for Grade C, and 0.883 for Grade D, giving a macro-averaged AUC of 0.871 (Table 1 and Table 2). Discrimination was strongest for the two terminal grades and weaker for the intermediate grades, consistent with the confusion matrix. Because mastication quality is continuous, samples near adjacent grade boundaries may have very similar MIT features, which limits precise classification. The macro-averaged AUCs of XGBoost (0.861) and SVM (0.847) were slightly lower than that of RF by no more than 0.024.

#### 3.4.3. Practical Value Assessment of Grade Classification

The RF accuracy of 64.3% and AUC of 0.871 are not yet sufficient for industrial four-grade sorting. Nevertheless, the higher recognition rates for Grades A and D (84.0% and 64.0%) may support preliminary identification of upper-quality and low-quality fruit. The high confusion rate between Grades B and C would require confidence thresholds and manual reinspection. In commercial grading, an error between adjacent grades is generally less consequential than an error spanning multiple grades because it produces a smaller pricing discrepancy. The low frequency of errors spanning two or more grades therefore provides a useful basis for further improvement through larger datasets and refined feature engineering.

## 4. Discussion

### 4.1. Physical Interpretation of Frequency Band Sensitivity

The results of this study suggest that the mid-to-high frequency band (22–29 MHz) of the MIT signal may contribute more to the characterization of citrus mastication-related targets than the low-frequency band. This pattern could be related to the frequency-dependent dielectric properties of biological tissues. According to the Cole–Cole dielectric relaxation model [25], effective conductivity and dielectric constant can change with frequency. In the low-frequency band (15–21 MHz), the response may be dominated by bulk conductive pathways, whereas the mid-to-high frequency range may provide greater sensitivity to dielectric contrasts associated with segment membranes and juice sacs. Citrus segment membranes contain cellulose–pectin composite cell walls and generally have lower water content than juice-filled sacs [3,26], and reported differences in membrane organization between fruit with different mastication quality may also be relevant [2]. Differences in tissue composition and structure may therefore contribute to the observed concentration of selected MIT features in the mid-to-high frequency range, although the present study did not directly measure tissue dielectric parameters.

The broader frequency interval associated with shear force may reflect the combined contribution of segment-membrane fiber bundles and internal vascular bundles during shear testing [26]. The broader frequency range could therefore be related to the more complex mechanical response measured by shear force. These interpretations remain tentative and should be tested with tissue-level dielectric measurements and independent validation [23].

The reduced importance of 15–21 MHz signals is consistent with reports by Griffiths et al. [12] and Watson et al. [27] on MIT responses in biological soft tissues. At lower frequencies, eddy currents may mainly reflect bulk conductivity, which can provide limited sensitivity to fine fibrous structures. Spatial importance was concentrated near the equatorial section, where segment interfaces occupy a substantial portion of the measured transverse plane. This anatomical correspondence is consistent with the proposed sensitivity of MIT to internal citrus tissue structure, but the present analysis did not directly register the signal map with histological features [14].

### 4.2. Comparative Analysis of Model Prediction Performance

In this study, the best task-specific results were obtained by SVM for puncture force (R^2^ = 0.825) and shear force (R^2^ = 0.762), RF for chewiness (R^2^ = 0.849), and XGBoost for the comprehensive mastication score (R^2^ = 0.835; RRMSE = 0.302). These results provide a task-specific benchmark for MIT-based prediction of citrus mastication quality traits. Previous studies have reported that near-infrared (NIR) spectroscopy combined with partial least squares regression (PLSR) typically yields R^2^ values in the range of 0.85–0.92 for the prediction of soluble solids content in citrus [7]; however, the predictive capability of NIR for internal mechanical texture indices is notably inferior to that for chemical composition indices, with R^2^ values generally below 0.75 [28]. Meanwhile, electrical impedance spectroscopy combined with support vector machine regression (SVR) has been reported to achieve an R^2^ of approximately 0.78–0.83 for predicting peach flesh firmness [29]. Direct cross-study comparisons should be interpreted cautiously because the targets, datasets, instruments, and validation designs differ. MIT nevertheless offers the practical advantage of non-contact measurement without coupling media.

As described in Section 3.2, the three models exhibited comparable performance, with SVM demonstrating a slight advantage in the regression tasks for puncture force and shear force. This phenomenon arises from the interaction between model characteristics and data conditions and can be interpreted from three perspectives. First, following PCA-based dimensionality reduction in this study, the feature dimensionality has been substantially compressed, and feature redundancy and noise have been effectively controlled. Under these conditions, the RBF kernel of SVM is able to fully exploit its capacity for constructing decision boundaries in high-dimensional space through nonlinear mapping [30], whereas the gradient boosting advantage of XGBoost yields relatively limited gains on dimensionality-reduced features with a moderate sample size (*n* = 300). Second, the built-in L1 and L2 regularization of XGBoost provides favorable generalization capability at a sample size of 300; however, the piecewise fitting nature of tree-based models renders their performance in the fine-grained prediction of continuous target variables (e.g., chewiness) comparable to that of kernel-based methods. Third, the bagging strategy of random forest confers strong robustness to noise, achieving the highest R^2^ (0.849) among the three models for chewiness prediction. Nevertheless, the difference in prediction accuracy among the three models for the chewiness task is less than 0.01, indicating that chewiness, as a composite texture index, presents comparable modeling difficulty for both tree-based and kernel-based methods given the nonlinear structure of its MIT signal features. The distinct cluster at high forces in Figure 4A,B (low scores in Figure 4D) forms a subpopulation of roughing-disordered fruits, but the R^2^ of the predictions without the cluster drops less than 0.04 compared with that of all fruits, indicating the R^2^ inflation caused by the separated subpopulation is limited. In summary, all three models can effectively model associations between MIT signals and mastication indices under the conditions of this study; subsequent analyses will be discussed based on the overall performance of each model.

Because the mastication quality grade (GRADE) was derived from the comprehensive mastication score (SCORE) by equal-quartile binning, the two outputs share the same underlying quality construct but represent it differently. These grades represent relative positions within the score distribution of the study sample, rather than independently established or externally validated quality classes. Accordingly, the classification analysis evaluates the ability of MIT-derived features and machine-learning models to distinguish score-derived categories, not to validate predefined commercial or sensory quality standards. SCORE regression preserves continuous variation, whereas GRADE classification imposes fixed quartile boundaries. Samples close to a boundary may therefore be assigned to adjacent grades despite having similar mastication properties, which can make intermediate-grade discrimination more difficult. The results support using the continuous score as the primary output and the grade as a supplementary categorical interpretation, particularly when decisions involve intermediate-quality fruit.

### 4.3. Implications of Feature Overlap and Sensor Design Insights

The feature overlap pattern revealed by the UpSet plot analysis has implications for the optimization design of MIT sensors. The substantial overlap in selected features between CHEW and SF may imply that the two mechanical targets share some informative MIT-derived features. SF and PF co-occurred in 510 selected features (47.4% of each target-specific set), of which 103 were exclusive to the SF∩PF intersection and were not shared with the other three targets. Their respective exclusive features still accounted for substantial proportions (SF-exclusive: 223, 20.7%; PF-exclusive: 426, 39.6%), reflecting differences between the two measurement modalities. The comprehensive mastication score and GRADE shared 963 features (89.6% of their respective sets), constituting the largest intersection. GRADE possessed only 15 exclusive features (1.4% of its set; 0.6% of the total selected-feature pool), while SCORE possessed 63 (5.9%), indicating that the regression and classification tasks tended to converge in their selected feature subsets.

Because the informative features for puncture and shear force were concentrated within 22–32 MHz, reduced frequency sets may retain much of the information in the full 21-frequency protocol. However, CHEW had a high proportion of unique features, and SF had a broader characteristic range than the other targets. Frequency reduction could therefore decrease performance for these two variables. GRADE-specific features accounted for only 0.6% of the selected pool, suggesting that regression and classification may require similar frequency coverage. Systematic ablation studies should test alternative frequency subsets and quantify task-specific losses before a reduced-frequency sensor is designed [15,31].

### 4.4. Analysis of Practical Application Potential

Under the current protocol, the MT-200 requires 2–3 min per fruit and processes fewer than 30 fruit h^−1^, far below the throughput of commercial grading lines. This limitation prevents near-term integration into production-line sorting. The system may nevertheless have more immediate value as a phenotyping tool.

Rapid acquisition of mastication-quality phenotypes remains a bottleneck in citrus breeding and germplasm evaluation. Conventional texture analysis requires fruit segmentation and repeated puncture, compression and shear tests, taking approximately 10–15 min per fruit and requiring trained operators [5]. Sensory panels require substantial preparation and calibration, and their results can vary with panelist condition and experience [4]. Both methods are also destructive, so tested samples lose commercial value and may not remain available for subsequent observations or seed recovery. This is especially problematic when only a few fruits are available from an individual breeding line. MIT, by contrast, leaves the fruit intact, allowing the samples to remain available for commercial use, subsequent non-destructive observations, or seed recovery where applicable.

Although the MT-200 does not approach commercial sorting speed, it may improve efficiency in breeding trials that assess tens to hundreds of fruits per line. MIT can measure such a batch within several hours, whereas texture analysis may require several days and sensory panels typically evaluate no more than 20–30 samples per day. MIT also produces standardized digital phenotypes. Each measurement yields 21,504 frequency–spatial features that can be converted by the model into a continuous 0–10 score. This approach reduces variation among evaluators and batches and provides reproducible data for selection. With appropriate calibration and external validation, a common model may also improve comparison across locations and years, which is difficult to achieve with conventional sensory evaluation.

At present, MIT is therefore better positioned as a rapid phenotyping tool for citrus mastication quality than as an in-line sorter. In the near term, it could support small-batch evaluation in breeding stations and research institutions while preserving valuable plant material. In the medium term, multi-channel hardware optimization and standardized acquisition protocols could increase throughput for regional variety trials. In the longer term, miniaturized sensors and edge-computing modules may move the system toward production-line speeds. This staged pathway, from breeding phenotyping to in-line grading, acknowledges the current throughput gap while building on the non-destructive, objective, and standardized nature of MIT.

### 4.5. Limitations of the Study

This study has the following limitations that warrant attention in future research.

First, hardware generalizability is uncertain. All experiments used the MT-200 system, whose coil geometry, frequency coverage, and acquisition protocol are instrument-specific. The transferability of the feature pipeline and predictive models to other MIT platforms has not been tested. Differences in sensitivity, frequency resolution, and spatial reconstruction may alter feature distributions and prediction accuracy. Cross-device validation and transfer-learning studies are needed to assess hardware independence and support broader application [32].

Second, the study included only ‘Miyagawa’ satsuma mandarin (*Citrus unshiu Marcov.*). Other cultivars, including Ponkan, Orah, and navel orange, differ in fruit size, segment morphology, membrane thickness, and juice-sac structure. These differences may affect MIT frequency–spatial responses [33]. Model performance should therefore be evaluated across multiple cultivars. Additional variation may arise from production region, orchard management, and harvest timing. Future studies should include multiple regions, cultivars, and even fruit size and peel thickness to develop models with stronger external validity.

Third, AHP weight assignment is partly subjective. The comprehensive score combined texture variables and sensory evaluations using weights derived from expert judgments. Different expert panels or consumer markets may assign different importance to puncture force, shear force, chewiness, and sensory palatability, which would affect score values and grade boundaries. Larger consumer-preference studies could be used to estimate weights statistically. Sensory evaluation using alternative weighting schemes would also help quantify the effect of weight uncertainty on model stability.

Fourth, the mechanical reference measurements do not fully characterize the intrinsic mechanical properties of citrus tissues. The target measurements in the present study do not directly quantify an intrinsic material property such as the modulus of elasticity, which limits the interpretation of the predictions as estimates of fundamental tissue mechanics. Combining these measures is a possible approach to improve the prediction of citrus mastication quality in the future.

## 5. Conclusions

This study presents a proof-of-concept application of magnetic induction tomography (MIT) to the non-destructive assessment of citrus mastication quality. Signals at 22–29 MHz contributed most strongly, and selected features were concentrated near the equatorial section. These patterns may link the frequency–spatial MIT response to segment membrane–juice sac structure and provide a basis for selecting sensor frequencies. Based on pooled out-of-fold predictions, SVM achieved the highest R^2^ for puncture force (0.825) and shear force (0.762), RF achieved the highest R^2^ for chewiness (0.849) and the highest grade-classification accuracy (64.3%) and macro-averaged AUC (0.871), and XGBoost achieved the highest R^2^ for the comprehensive mastication score (0.835). The continuous score retains information that can be obscured by rigid grade boundaries, particularly for intermediate-quality fruit. The three models showed similar performance for chewiness, so future work should test whether their errors are sufficiently complementary to justify an ensemble approach. Although current throughput (<30 fruit h^−1^) is insufficient for commercial grading lines, MIT shows promise as a non-destructive, objective, and standardized phenotyping tool. Measured fruit can remain available for commercial use, subsequent non-destructive observations, or seed recovery where applicable, and batch measurement is faster than conventional texture and sensory protocols. However, because this study was limited to ‘Miyagawa’ satsuma mandarins collected from a single orchard, the generalizability of the proposed approach to other citrus species and cultivars has not yet been established. Future studies should validate the approach across diverse citrus types and production conditions and its applicability to in-line grading.

## Figures and Tables

**Table 1:** Multi-task prediction performance evaluation of XGBoost, Random Forest, and SVM based on 10-fold cross-validation.

Model		XGBoost	Random Forest	SVM
Puncture force	R^2^	0.789	0.816	0.825
	RMSE	30.992	29.006	28.211
	RRMSE	0.156	0.146	0.142
Shear force	R^2^	0.729	0.759	0.762
	RMSE	35.783	33.678	33.444
	RRMSE	0.153	0.144	0.143
Chewiness	R^2^	0.842	0.849	0.846
	RMSE	2.781	2.722	2.746
	RRMSE	0.235	0.23	0.232
Mastication Score	R^2^	0.835	0.83	0.785
	RMSE	1.029	1.043	1.108
	RRMSE	0.156	0.158	0.168
Grade	Accuracy	0.617	0.643	0.633
	AUC	0.861	0.871	0.847

**Table 2:** Classification performance comparison of three machine learning models for mastication quality grade assessment (one-versus-rest AUC, outer 10-fold cross-validation).

Model	A		B		C		D	
	AUC	Accuracy	AUC	Accuracy	AUC	Accuracy	AUC	Accuracy
XGBoost	0.950	0.827	0.813	0.520	0.789	0.453	0.894	0.667
Random Forest	0.958	0.840	0.828	0.560	0.813	0.533	0.883	0.640
SVM	0.951	0.880	0.792	0.560	0.785	0.573	0.859	0.520

## Data Availability

The original contributions presented in this study are included in the article material. Further inquiries can be directed to the corresponding author(s).

## Supplementary Materials

The following supporting information can be downloaded at https://www.mdpi.com/article/10.3390/plants15213276/s1, Table S1: SNR of the MIT system under each frequency; Table S2: Instrumental texture measurements and fruit diameter of ‘Miyagawa’ satsuma mandarin fruit samples; Table S3. AHP-derived weights results of comprehensive mastication score.
